# Characterization of the complete chloroplast genome of a medicinal species *Euodia ruticarpa* in China (Rutaceae)

**DOI:** 10.1080/23802359.2020.1829125

**Published:** 2020-10-12

**Authors:** Hao Liu, Jian Jin, Rongrong Zhou, Can Zhong, Jing Xie, Weiliang Zhou, Shuihan Zhang

**Affiliations:** a Hunan Academy of Chinese Medicine, Institute of Chinese Materia Medica, Changsha, PR China; b Hunan University of Chinese Medicine, Changsha, PR China; c Chinese Academy of Chinese Medical Sciences, National Resource Center for Chinese Meteria Medica, Beijing, PR China; d Hunan Shenzhou Chinese Medicine Inc, Cili, China

**Keywords:** *Euodia ruticarpa*, high-throughput sequencing, chloroplast, genome sequence

## Abstract

*Euodia ruticarpa* is a medicinal plant recorded in in Chinese Pharmacopeia. Here we report on the complete chloroplast genome sequence of *Euodia ruticarpa*. The chloroplast genome is 158,762 bp in size and includes two inverted repeat regions of 54,230 bp, which is separated by a large single-copy region of 86,267 bp and a small single copy region of 18,265 bp. A total of 131 genes were predicted, including 37 tRNA, 8 rRNA, and 86 protein-coding genes. Phylogenetic analysis placed *Euodia ruticarpa* under the family Rutaceae.

*Euodia ruticarpa* (synonym: *Tetradium ruticarpum)*, recorded in in Chinese Pharmacopeia, is one of the well-known medicinal herbs in China. The dried unripe fruit of *Euodia ruticarpa* known as ‘Wuzhuyu’ is traditionally and ethnically used as crude medicine drug (Zhao et al. 2019). In traditional Chinese medicine, Wuzhuyu could be used either alone or in combination with other herbal medicines to cure a lot of diseases, such as headache, epigastric pain, menorrhalgia, dermatophytosis, emesis and aphtha (Tian et al. 2019). Although the chemical constituents (Zhou et al. 2010), anti-inflammatory effects (Liao et al. 2011) and complete chloroplast genome of its variant *Euodia ruticarpa* var. *bodinieri* (Liu et al. 2020) were ever reported, available genetic resource currently for *Euodia ruticarpa* is still limited. Therefore, it is necessary to develop the genetic resources to further investigate the germplasm of this species.

In this study, we aimed to characterize the complete cp genome sequence of *Euodia ruticarpa* to serve as a valuable genomic resource. Total genomic DNA was extracted from fresh leaves of *Euodia ruticarpa* by CTAB method (Mcpherson et al. 2013), planted in Botanical Garden, Anhui University of Chinese Medicine (N31°56′34.74″, E112°23′01.77″). Additional leaf specimens were kept in Hunan Herbarium of Chinese Traditional Medicine under the collection number HUTM100006.

NEBNext Ultra DNA Library Prep Kit (Illumina, USA) was used to construct a genomic library consisting of an insert size of 350 bp. Sequencing was carried out on an Illumina NovaSeq platform. The output was a 7.0 Gb raw data of 150 bp paired-end reads, further trimmed and assembled using SPAdes (Bankevich et al. 2012). Annotations of chloroplast genome were conducted by the GeSeq website and software PGA (Plastid Genome Annotator) (Qu et al. 2019), and checked by comparison against the *Euodia ruticarpa* var. *bodinieri* complete chloroplast genome (GenBank accession number: MT134114) (Liu et al. 2020).

The complete chloroplast genome of *Euodia ruticarpa* (GenBank accession number: MT 800757) is 158,762 bp in length, displaying a quadripartite structure that contains a pair of inverted repeats (IR) regions (54,230 bp, GC content 42.82%), separated by a large single-copy (LSC) region (86,267 bp, GC content 36.61%) and a small single-copy (SSC) region (18,265 bp, GC content 33.16%). There are 131 genes reported, including 8 rRNA genes, 37 rRNA genes, and 86 protein-coding genes. The overall GC content of the cp genome was 38.33%.

For phylogenetic analysis, a maximum-likelihood tree was constructed with 1000 bootstrap replicates using FastTree software (Liu et al. 2011). A subset of another 17 species from the family Rutaceae was included, with *Toona ciliata* from Meliaceae as outgroup. FastTree uses Generalized Time-Reversible (GTR), the best-fitting model of nucleotide substitution models, for nucleotide evolution analysis. As shown in Figure 1, *Euodia ruticarpa* is placed under the family Rutaceae. The taxonomic status of *Euodia ruticarpa* exhibits a closest relationship with its variant *Euodia ruticarpa* var. *bodinieri*. This finding could provide insight into conservation, exploitation and genetic evolution for this medicinal plant species.

**Figure 1.**
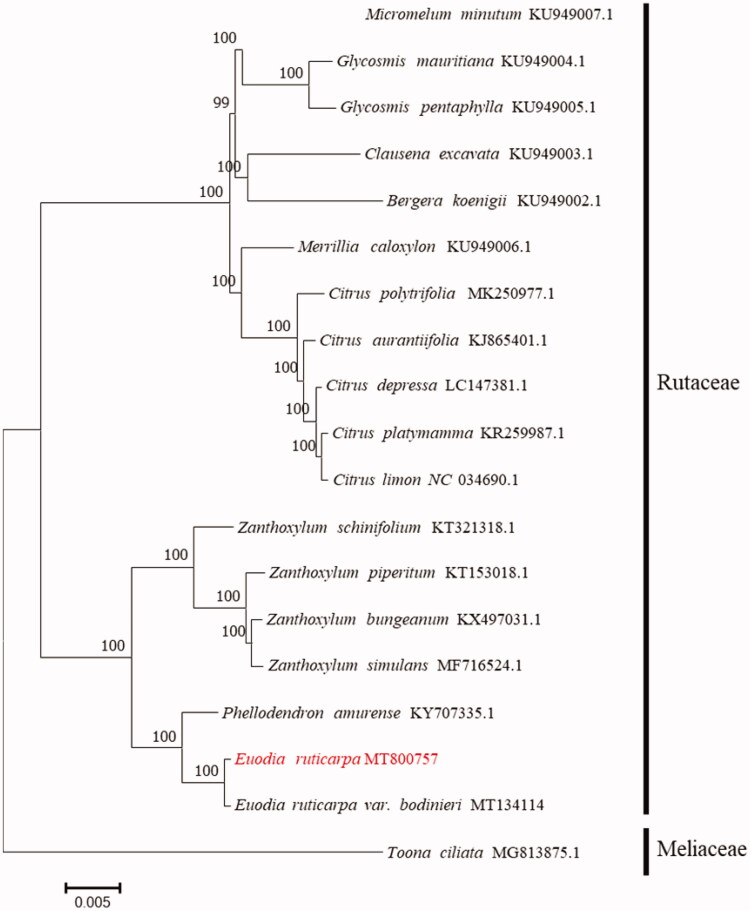
Maximum-likelihood tree based on the complete chloroplast genome sequences of 17 species from the family Rutaceae with *Toona ciliata* from Meliaceae as outgroup. The bootstrap values were based on 1000 replicates.

## Data Availability

The data that support the findings of this study are openly available in GenBank of NCBI at https://www.ncbi.nlm.nih.gov, reference number MT 800757.
